# Affections of the Chest

**Published:** 1829-04

**Authors:** 


### AFFECTIONS OF THE CHEST.

*Cases of Affections of the Chest, succeeding Operations and Injuries, treated at the* Glasgow Royal Infirmary.

Case I.—David M'Lardy, æt. twenty-one, was admitted December 29th, with a small fistula on right side of anus, discharging a little thin matter, but not communicating with the gut. It commenced four years ago, and was supposed to be nearly healed, when, five months since, the discharge was renewed, without any pain. The fistula was cut in the usual way on the 4th of January. On the 6th, he complained of griping pains. On the 7th, he had a rigor, followed by severe pain of the back, extending to the testicles. He was feverish and uneasy; pulse ninety; tongue whitish; thirst; urine high coloured. His bowels had been opened by castor oil, and a poultice was applied to the wound.

On the morning of the 10th, the feverish symptoms continuing, he was bled to sixteen ounces. Blood buffy. Complains chiefly of pain in testicles; edges of the wound feel hard; pulse ninety-six; bowels open. In the evening, had six grains of calomel, with one of opium, and the hip bath; which was followed by profuse perspiration and great relief from pain.

December 11th.—Complains chiefly of weakness; pulse eighty; thirst.—Pulv. Doveri gr. xij. vespere.

13th.—Had sickness and vomiting last night. Hip bath this morning, which produced copious perspiration. A slight rigor during the forenoon; pain of back and testicles; lower part of abdomen slightly tympanitic, but not painful; bowels rather slow; pulse ninety-four; tongue cleaner.—Had twelve leeches to abdomen, and a purging enema.

On the 18th, an abscess which had formed on left hip was punctured, and four ounces of matter evacuated. A healthy purulent discharge from the hip continued, but the feverish symptoms did not abate; and on the 21st another abscess, near the former, containing half an ounce of pus, was opened. During the night he had some delirium, and next day complained of pain of abdomen, which was much distended and tympanitic. Countenance sunk; pulse 120. Symptoms continued with little change till the 25th, when he had frequent cough, with expectoration containing some blood. On examination by the stethoscope, the right lung was found to be consolidated. Had a blister to the breast, and the Vinum Antimon. He sunk without any aggravation of symptoms, and died on the 28th.

*Dissection.*—The abscesses on the hip much contracted, and contained no pus. The colon was greatly distended with air, but the texture of that, and of all the other abdominal viscera, seemed free from disease. The right lung was found adhering extensively to the sides of the thorax, and nearly the whole of this lung was changed in structure: it was mostly in a state of hepatization, but in some parts was beginning to pass into that of purulent infiltration. The left lung was perfectly healthy.

This case is an interesting example of the insidious manner in which affections of the chest frequently succeed operations. Until three days before death, no suspicion appears to have been entertained of any pulmonary disease: all the symptoms pointed to the abdomen and pelvis. When the formation of abscesses over the hip did not remove the fever, it might reasonably be concluded that some mischief was going on in the cavity of the abdomen: probably the formation of matter within the pelvis. Slight cough and puro-mucous expectoration were the first and only symptoms of chest affection. Previously he had been repeatedly questioned as to whether he had cough, or any uneasiness about the breast, but he always affirmed he had not; and it was, we presume, owing to the absence of all symptoms denoting an affection of the lungs that a more rigid examination was not earlier had recourse to. The knowledge at length obtained by means of the stethoscope, if it ever could have availed to save his life, came too late to be of any service.

Case II.—Helen Blackie, æt. forty, was admitted on the 2d of January. The day before she had fallen down several steps of a stone stair, and had received a wound of the scalp, extending from near the lambdoidal suture two and a half inches forwards, and then transversely across right side of the head for about five inches. The edges of the wound had been brought together by stitches, and a considerable part had adhered; but in one or two points the probe passed several inches backwards and forwards, and a considerable quantity of thin fetid pus was discharged. She had received, at the same time, an injury on the left side of the chest, which through its whole extent was emphysematous, but no fractured ribs were detected. Much pain was felt, and increased on deep inspiration, at lower anterior part of chest. Had difficulty of breathing, headach, and tinnitus aurium, with dulness of hearing; thirst; skin hot; tongue white; bowels costive; pulse 100. Profuse suppuration and disunion of the wound followed; and in a few days after admission the pericranium was found destroyed to some extent. The patient became weak, and was allowed a small quantity of wine; after which she began to recruit, and the suppuration rapidly diminished. The emphysema, dyspnœa, and pain of chest, disappeared after wearing the thorax bandage. She had slight, but not troublesome cough.

She went on favorably until the 29th, when she became uneasy and fretful, was averse to being disturbed, and her deafness increased. She was feverish, and her pulse had risen to ninety-six; but she complained only of weakness. The wine was omitted, and she was ordered a purge.

On the 30th, her bowels were freely opened, and the pulse had sunk to seventy-six: she still, however, seemed uneasy, but made no positive complaint.

On the 2d of February the report is, discharge from scalp increasing and thin; has cough, with expectoration and slight dyspnœa; pulse 100; tongue moist; bowels open.—℞. Submur. Hydrarg. gr. vi.; Pulv. Antim. ℈i.; Pulv. Opii gr. v. M. div. in vi. 1 ter indies.

On the 3d, features were shrunk, and she seemed fast sinking. Pupils contracted. Complained of no pain, and cough was less troublesome; pulse 100. She died on the morning of the 4th.

*Dissection.*—A considerable part of wound of scalp remained disunited. A portion of bone, about three inches square, was denuded of its pericranium. The corresponding part of the inner table was rough, and of a more yellow colour than natural. Beneath it the dura mater was slightly thickened, vascular, and covered with pus. A small abscess had formed in cerebral substance, at superior part of the right middle lobe of the brain: it contained greenish yellow pus. The brain was firm, and its blood-vessels distended. A very small quantity of fluid was found in the ventricles. In the left side of the thorax several ounces of sero-purulent fluid were found: the lung was covered with coagulable lymph, a third of it was in a state approaching to hepatization, and several small portions of its substance were indurated.

These portions varied in size from a small hazel nut to a walnut, and were seated immediately under the pleura. Over two or three of them this membrane was of a dirty yellow colour, and, when they were cut into, pus oozed freely from the cut surface: from most of the others purulent matter could be pressed. In the opposite lung were four or five points of a similar nature.

